# Supplementary figures and images for: Impact of tps1 Deletion and Overexpression on Terpene Metabolites in Trichoderma atroviride
Source: J Fungi (Basel). 2024 Jul 14;10(7):485. doi: 10.3390/jof10070485 (PMC11278490; doi:10.3390/jof10070485)

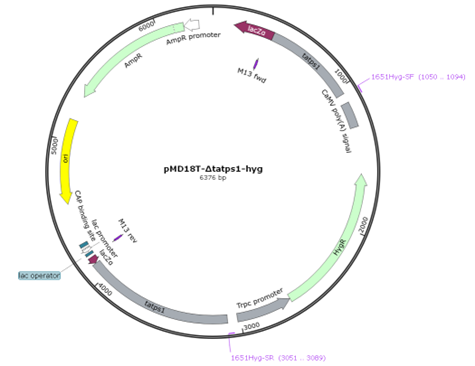

Supplement: Supplementary file 1 [file jof-10-00485-s001.zip › jof-3070127-Figure S1.png]
